# Tailored support for preparing employees with cancer to return to work: Recognition and gaining new insights in an open atmosphere

**DOI:** 10.3233/WOR-220566

**Published:** 2023-12-15

**Authors:** Corine M. Tiedtke, Roland W.B. Blonk, Willem Van Rhenen, Martine P. Van Egmond, Margot C.W. Joosen

**Affiliations:** a Tranzo-Scientific Center for Care and Wellbeing, Tilburg School of Social and Behavioral Sciences, Tilburg University, Tilburg, The Netherlands; b Department of Human Resource Studies, Tilburg School of Social and Behavioral Sciences, Tilburg University, Tilburg, The Netherlands; c TNO Innovation for Life, Leiden, The Netherlands; d Optentia Research Unit, North-West University, Vanderbijlpark, South Africa; e Arbo Unie, Nieuwegein, The Netherlands; f Center for Human Resource Organization and Management Effectiveness, Nyenrode Business University, Breukelen, The Netherlands

**Keywords:** Oncology, work participation, human-centered approach

## Abstract

**BACKGROUND:**

A considerable number of cancer survivors face difficulties in returning to work (RTW). More insight is needed on how to support employees shortly after cancer treatment and help them make the transition back to work.

**OBJECTIVE:**

To gain an in-depth understanding of how and under what circumstances a Cancer & Work Support (CWS) program, which assists sick-listed employees with cancer in preparing their RTW, works.

**METHODS:**

A qualitative design was used, inspired by Grounded Theory and Realist Evaluation components. Semi-structured interviews were conducted with RTW professionals (*N* = 8) and employees with cancer (*N* = 14). Interview themes covered experiences with CWS, active elements, and impeding and facilitating factors. Interviews were transcribed and analyzed by multiple researchers for contextual factors, active mechanisms, and the outcomes experienced.

**RESULTS:**

Respondents experienced the support as human centered, identifying two characteristics: ‘Involvement’ (‘how’ the support was offered), and ‘Approach’ (‘what’ was offered). Four themes were perceived as important active elements: 1) open connection and communication, 2) recognition and attention, 3) guiding awareness and reflection, and 4) providing strategies for coping with the situation. Variation in the experiences and RTW outcomes, appeared to be related to the personal, medical and environmental context.

**CONCLUSION:**

Both professionals and employees really appreciated the CWS because it contributed to RTW after cancer. This research shows that not only ‘what’ RTW professionals do, but also ‘how’ they do it, is important for meaningful RTW support. A good relationship in an open and understanding atmosphere can contribute to the receptiveness (of employees) for cancer support.

## 1 Introduction

In Europe, cancer has increased to more than 3.5 million new cases and nearly 2 million deaths each year [1]. Knowing that many of the newly diagnosed cancer patients are of working age, facilitating return-to-work (RTW) after cancer should be encouraged [2, 3] but without pressure [4]. The literature shows that employees diagnosed with cancer are eager to return to normality and leave behind the sick role, and this includes going back to work [5]. Returning to work has additional benefits: it can be a distraction from the illness, meet financial needs, improve quality of life and reinstall a survivor’s identity. However, resuming work can be challenging because of the physical and cognitive side effects that are experienced [6]. Psycho-educational support is essential to facilitate RTW [7]. In addition, cancer survivors may feel uncertain and vulnerable or lack self-confidence about RTW [8].

It is well known that RTW rates after cancer can vary according to cancer type, treatment and duration of absence. Also, high demands at work and lack of (social) support can diminish the chances of successful RTW. Supportive measures are therefore required. In their review, De Boer et al. [9, 10] distinguished several types of supportive interventions: psycho-educational, vocational, physical, medical and multidisciplinary, with different impacts on RTW. They found that multidisciplinary interventions could enhance RTW of patients with cancer, whereas the outcomes of psycho-educational and vocational interventions are as yet unclear [9, 10]. However, good practices for supporting workability after cancer are scarcely known [11]. Recently, Stehle and colleagues [12] reported insufficient evidence to recommend occupational therapy interventions. Also, Algeo et al. [13] pointed at the lack of work-focused interventions to support RTW for women suffering from breast cancer.

Qualitative research is needed to better understand how RTW support is experienced in more detail during the different phases of the RTW process. Moreover, to obtain clear information on what should be discussed during the phases after treatment. For instance, when to talk about RTW with a cancer patient/survivor, and when to involve the employer. Previously, a qualitative study yielded that employees with cancer perceived their work absence due to cancer treatment in different ways. While absent from work, cancer survivors mentally prepared their RTW, considering how to become a worker again instead of being a patient. Furthermore, they reflected on their capability, based on their medical situation, and on the support to expect from the workplace [10]. Employers seem to play a key role in supporting the return-to-work (RTW) of their employees and in creating a good working and customized environment. Concurrently, they need support regarding information on cancer, communication with the employee, and arranging adaptations at work [14].

Depending on a country’s legislation, employers are obliged to collaborate with an occupational physician regarding RTW. With the Dutch legal requirements in mind, and in cooperation with a National Occupational Health and Safety Service, a supportive method called ‘Cancer & Work Support’ (CWS) was developed and tested, to support (preparing) the return to work of sick-listed employees with cancer. RTW professionals (i.e. social workers and reintegration coaches) offered the CWS to employees directly after cancer treatment. The CWS included three potential and theoretically founded modules: 1) Disease coping, 2) Skills/competences and 3) Resource management. More information on the support provided is given in the Methods section.

The CWS method was based on positive experiences of the JOBS program [15], which was applied in several groups experiencing ‘transition in life’ [16]. The principle of change in this transition (underlying the JOBS program) is creating mastery experiences thereby enhancing self-efficacy and improving the ability to deal with obstacles and setbacks [17].

The current qualitative study aims to gain an in-depth understanding from the existing method (Cancer & Work Support) to support sick-listed employees with cancer in preparing their RTW. Gathering knowledge on the experiences with the CWS can help professionals to understand the care and support needs of employees with cancer [18, 19]. The question to explore is: How do employees with cancer (receivers) and RTW professionals (deliverers) experience the support provided, regarding (preparing) RTW after cancer? In particular: when and how does the support provided work for employees/professionals?

## 2 Methods

### 2.1 Design

Using a qualitative design, semi-structured interviews with healthcare professionals, i.e. social workers and reintegration professionals (*N* = 8) and employees with cancer (*N* = 14), were conducted and thematically analyzed [20]. The design was inspired by Grounded Theory (GT) using the Qualitative Analysis Guide of Leuven (QUAGOL) [21] and Realist Evaluation (RE) components (searching for contexts, mechanisms and outcomes, yet not looking for causal explanations, since our aim was not to evaluate the CWS as an intervention, but to know when and how the CWS worked) [18, 19].

### 2.2 Ethical considerations

The medical ethical committee Brabant approved the study (NL63659.028.17 / P1756) and–because of the online interviewing –accepted an informed consent by mail, including name, date of birth and address of the participant. Anonymity of the participants was preserved in the Results section.

### 2.3 Context

In the Netherlands, employers have a contract with an occupational health and safety service. They are obliged to support the return-to-work (RTW) for two years, in collaboration with the occupational physician [22]. Instead of paying social premiums for sickness absence benefits, employers have to provide payment (at least 70% of the income) during these years. Then, the Employee Insurance Agency (EIA) for disability benefits, will assess the employee, taking into account the efforts made by both stakeholders regarding reintegration. If both the employee and the employer have done enough to achieve RTW, disability pension will be paid by the EIA.

### 2.4 Cancer & work support

The supportive method was tried out in several regional Dutch Occupational Health and Safety Services. Process coordinators were involved in the recruitment of participants for the study: i.e. RTW professionals (social workers and reintegration coaches) and sick-listed employees who delivered/received the support. Initially, occupational physicians informed their sick-listed employees about the existing method and employees were free to participate.

**Fig. 1:**
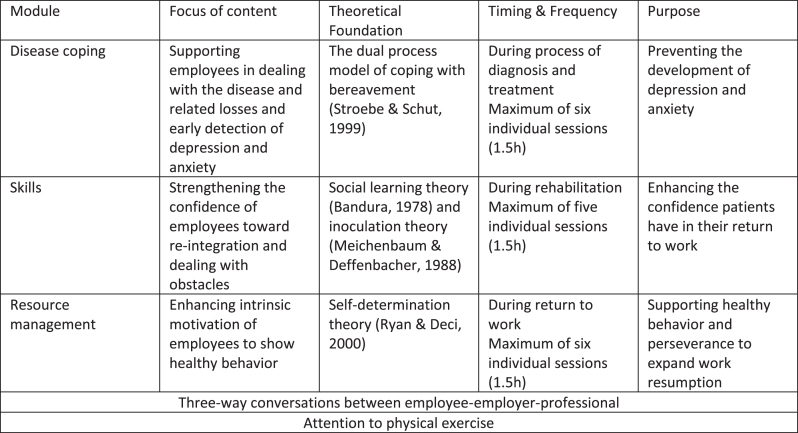
Cancer & work support.

As mentioned in the introduction, the CWS included three (potential) modules. The ‘Disease coping’ module was based on the dual process model of coping [23, 24]. The ‘Skills’ module was based on the social learning theory of Bandura [25] and inoculation theory of Meichenbaum and Deffenbacher [26]; and the ‘Resource management’ module on the Self-Determination Theory by Deci & Ryan [27]. A maximum of six sessions for each module was proposed. Within every session, physical exercise was a subject. Conversations with the employer were also included. The activities in the sessions aimed to support workability and reduce fatigue and possible mental problems. The RTW professionals were trained in disease coping and skills protocols beforehand. See Fig. 1.

### 2.5 Data collection

Process coordinators of about 18 regional National Occupational Health and Safety Services (the Dutch ArboNed) invited RTW professionals (social workers and reintegration coaches), who had been carrying out different modules of the support: ‘disease coping’, ‘skills/competences’ and/or ‘resource management’, by an informed mail. Likewise, the supported employees with cancer were invited to participate, as well as those who were still involved in a module. After a few recalls, eight professionals and fourteen employees responded and were included in the study (convenience sample). An additional call was made to inform them again about the interview and to collect personal information (e.g. age, gender, diagnosis, occupation). Then they replied with an informed consent mail. In close consultation with the participants an appointment for the interviews was made. They determined the time and the form (video call/telephone interview). See Table 1.

**Table 1:** Participant characteristics

Employees	(N = 14)	Professionals	(N = 8)
*Age*		Age
30-40	2	30-40	2
40-50	6	40-50	1
50-60	4	50-60	2
>60	2	>60	3
*Gender*		*Gender*
Female	9	Female	7
Male	5	Male	1
*Cancer diagnosis*		*Position*
Breast	7	Social worker	5
Lymph node	3	Reintegration coach	3
Testicle	1
Large intestine	1
Prostate	1
Adrenal gland	1
*Working sector*		*Supported employees*
Engineering	2	One	3
Commerce	7	Two	2
Retail	3	Five	1
Medical	2	Ten	1
		Fifteen	1
*Departmental, Account, Branch Managers, Commercial and Medical Assistants, Work Foremen*		*Not automatically matched to the employees interviewed*
*Returned to work (during or after the support)*
Full-time	5
Part-time	7
No return	2

### 2.6 Interviews

Due to the Covid-19 virus, we could only conduct online interviews. To protect the participants’ privacy, we used MS-Teams/ZOOM H2 Handy Recorder for interviewing/recording and Express Scribe for transcribing the interviews, in consultation with the university’s IT service. A topic guide was developed to structure the interviews with both the employees and the professionals – see Appendix. Participants were asked how they looked back at the support and what the support yielded. The topics of the interviews included the frequency and timing of the different modules of the support program, strengths/weaknesses of the support (attuned to the phase you were in) and RTW experiences (employer contact, what has it given you). For the professionals we added questions on protocols and scope for action. We started with an introductory talk and a few general questions (do you know when you started the Cancer & Work Support and which modules were offered/followed then; what did you appreciate most and why?). Then, we continued to ask questions about what was of particular interest for the person concerned. During the interviews, we asked – in case of doubt – for reflection on what was said, so that we could get as clear a picture of the experiences as possible. Participants could choose whether to receive a voucher or to donate the small sum to the Dutch Cancer Society (KWF). The first author, an experienced qualitative researcher (CT), performed and fully transcribed the interviews. The interviews lasted on average 45 minutes. After the interviews and analysis, the participants received the results of the research/interview; we did not receive any response to the findings.

### 2.7 Analysis

Inspired by Grounded Theory, using the Qualitative Analysis Guide of Leuven (QUAGOL) [21] and Realist Evaluation components [18, 19], we tried to understand the support provided while labeling contexts, mechanisms and outcomes, yet not searching for causal connections. We used an open approach and did not use initially drawn-up theories and hypotheses, as we weren’t aiming to measure the effectiveness of the Cancer and Work Support (CWS). We focused on the (themes in the) mechanisms, as we were most interested in what exactly happened and was experienced during the sessions.

While studying the transcripts (reading with the research question in mind, as many times as necessary) and monitoring data-saturation (which might not be reachable considering the various characteristics of the participants), narrative and conceptual reports were made per interview (CT) [21]. At the same time, working mechanisms, contexts and outcomes were highlighted and coded in the transcripts by three authors independently of each other (CT, RB, MJ) [18, 19]. For all transcripts, and based on the conceptual reports, core messages and meaningful themes –derived from the contexts (about attitude, medical and work situation, environmental support), mechanisms (about communication, awareness, involvement, approach) and outcomes (return/no return after support) –were identified and listed (CT). In cooperation with the research team, these messages and themes were repeatedly and intensively discussed to be able to structure and describe the findings in a useful and logical way. Final decisions were made by consensus and in cooperation with all authors.

## 3 Results

Both receivers and providers characterized the Cancer & Work Support (CWS) as human centered. To be able to meet the employees’ needs and to adapt to the situation, the RTW professionals tailored the CWS. We distinguished two characteristics of the CWS: Involvement (regarding the form: ‘how’) and Approach (regarding the content: ‘what’). Four themes in total were covered: open connection and communication; recognition and attention; guiding awareness and reflection; providing strategies to deal with the situation. Variation in the experiences seemed to be related to the personal, medical and environmental context. Below, we first outlined how the support was tailored by the professionals. Next, the four themes of both characteristics and the different contexts were described. Finally, we considered the value of the CWS. While describing the findings, the experiences of the employees [E] and the RTW professionals [P] were integrated.

### 3.1 Tailored support

At the start of the support, which was in general the disease coping module, the professionals mentioned that they really wanted to adapt to the needs of the employee. As regards timing, they experienced however that the modules did not always harmonize with the employee’s phase of recovery. Individuals also seemed to differ regarding disease coping and progress. In close consultation with the employee, workable choices were made.

“*What are the care and support needs? That is determined together with that person. This is also much more in line with our method, connecting with the client. After that it was determined: which intervention should be used. [P2]*”

Throughout the sessions, this could lead to postponed or spread-out consultations (e.g. because of additional medical therapy), to choosing appropriate exercises (e.g. reflection tasks seemed less suitable for commonsensical doers), to advice to stop the coping module, or, to refer to the next module.

“*The proposed protocol was not always appropriate. Questions such as ‘how are you going to communicate what is going on to your environment and your employer*’*, had often been discussed already. [P4]*”

The interviewed professionals themselves (i.e. social workers, reintegration coaches) experienced that the support to be given was a nice and complete method, but with a large number of time-consuming exercises for the employee shortly after cancer treatment and a lot of preparation time for the professionals.

“*One minute before you bring someone in, you don*’*t have that program in your mind again. It really requires a lot more preparation (* . . . *). You have to know by heart, the choices that you can present to the person. [P3]*”

Professionalism and experience –being able to diverge from the prepared session –was found to be important for the RTW professionals. They frequently had to adapt the tight protocol, to let go of the structure and/or improvise, in order to meet the specific needs of their client and to stay in good contact (not to lose him/her).

### 3.2 Involvement

#### 3.2.1 Open connection and communication

The interviews showed that the employees really appreciated the support, although they did not fully remember the precise content of the sessions and the modules attended.

“*I have also received a number of assignments. I think I completed those properly every time, and while talking we also discussed them. But I wouldn’t know exactly what it all was* . . .  *[E11]*”

The atmosphere during the sessions seemed especially valuable. The participants felt that the professional was on their side, unlike the medical staff. Communication seemed to be more on the same level and topics could be addressed and worked out together. Almost everyone mentioned that there was a ‘click’ with the professional in question.

“*We just had a very good relationship, a good click, and she understood exactly what my problem was. [E13]*”

A great connection was felt gradually. All support was welcome. Even for those who felt they did not need support when they were invited to participate, it proved useful and pleasant to be able to put everything together with an objective and non-judgmental expert. One of the first experiences mentioned was that during the conversations they felt human again, like a searching individual.

“*It is nice to be able to tell your story and to get tips. To be heard by people who do not work in a hospital* –*and someone who is not the occupational physician. At that moment, you do not feel so much like the patient, but an individual who is looking to tie all the strings back together. [E3]*”

In particular, the employees remarked that they could freely tell their story to a professional who knew what she was talking about, and who acted as a permanent point of contact. Many said they received energy from the conversations and felt more at ease about their situation. This woman mentioned how she learned about communication and that she was more willing to get in touch with her employer again.

“*With the help of the first module, I managed to communicate with my employer and my colleagues. So that they could understand more about my situation and I more about their situation. [E3]*”

#### 3.2.2 Recognition and attention

Beyond the open atmosphere during the conversations, the attentive way the RTW professional treated the employees was highly appreciated. What stayed with them the most was that there was a ‘trusted’ someone who understands you, pays attention to you, thinks along with you, provides structure, motivates you and directs you; who confirms and recognizes you in the steps you take, who gives you space to discuss topics that affect you or that bother you. The employees felt able to get to the bottom of what was worrying or frightening them, whether it concerned work-related or private matters. They felt relief at being able to vent, expose their deepest inner self, to cry and laugh.

“*She has guided me in dealing with my fears. I am grateful that the occupational health service gave me the opportunity, that I had a social worker who kicked my butt. Where I was allowed to cry, where I could laugh, but who understood me, and also just held my hand for a moment like,* ‘*you are having a hard time*’*. I felt alone, I felt lonely. She pulled me through all of that. That’s great if you have someone who can do that for you. [E13]*”

As the data showed, you were allowed to be yourself and only think of yourself. Feeling that recognition, attention, empathy and concern made the employees feel especially safe. Being guided in this way the employees could think about their situation, their competences and then shape new priorities in peace and quiet.

### 3.3 Approach

#### 3.3.1 Guiding awareness and reflection

As the interviews revealed, the RTW professionals offered safety and confidence. One of the first things they did was to normalize the employees’ intense feelings.

“*I think normalization really is a task of the social worker (* . . . *) you have to know the difference. If it leans towards something psychiatric, you have to pay attention to it (...) People are often also afraid of the fear (...) Yes, that normalizing part, that can take away your fear. [P7]*”

The professionals continued to ask questions about how the employee felt, as a person and as a worker with cancer. The interviewed employees mentioned that the coaches cleared things up, structured the person’s stories and gave advice, after having listened carefully. They felt motivated in focusing and reflecting on feelings, decisions and actions.

“*That you feel heard with your complaints, that is perhaps the most important thing (...) but we also just give really useful tips. It is the combination of that listening ear* –*of someone who is really independent and knowledgeable and who understands you, who knows what it is about* –*and the practical tips. [P6]*”

Employees called this support strengthening and helpful in regaining self-confidence.

#### 3.3.2 Providing strategies to deal with the situation

From the interviews, we learned that the professionals were aware of the difficulties the employees faced shortly after treatment. They might feel mentally confused, being in a process of surviving. The professionals noticed that they were able to help the employees find a new or more stable way of life. The interviewed employees mentioned that they were frequently made aware of the need to manage their energy. Many examples were given of how to take enough rest and make time for relaxation. Useful tools, various instruments with exercises and concrete tips were given regarding managing the employees’ concerns, anxiety and pitfalls.

“*You know, there are just really good things in the module. They help to provide insight into who am I, what are my qualities, which obstacles do I encounter, which priorities do I have to set (...) yes, with lots of tips and tools, they could really get started. [P6]*”

Many employees said that they learned in this way how to cope with their feelings in different ways in order to accept their situation gradually.

“*Especially putting things into perspective. I can handle it in a more relaxed way. I have learned not to keep looking back to the past. [E2]*”

With regard to their work, realistic plans to return were built up, taking into account the person’s competence, ability and energy.

“*She was very clear with me:* ‘*how are we going to pick it up to return?*’ *Because I was really in the dark about that. Do I have to try again, return fulltime, and see what happens? She gave me very good tips there. [E9]*”

Enough time was given to map out one’s competences and establish new goals. The interviewed employees felt it also helped to explore potential new aspirations (a new study, a new job).

The support described above shows that the open atmosphere and the genuine attention was highly appreciated by the employees. Apparently, this was a good starting point for the professionals to work further with the employee in guiding awareness and providing strategies to deal with the personal and the work situation.

### 3.4 Context

Although both employees and RTW professionals very much appreciated the support received, respectively given, the experiences of the participants varied. This worker summarized the contextual factors regarding the support as follows:

“*In my case there were already many advantages such as that I have good prognoses. Besides, I have such a good relationship with everyone, with the owner of the company and with the manager. How I am as a person. That also plays a role in the reintegration. However, I do think it has helped that she has guided me a bit in listening to my body carefully, listening to my head carefully. Balancing energy. [E4]*”

#### 3.4.1 Personal differences (receivers/providers)

Irrespective of the support, attitudes towards the illness and work could differ. Some employees underlined their gloomy state of mind regarding the work situation or their wait-and-see attitude. Others mentioned their motivation or positive state of mind and their eagerness to proceed during the RTW process. A realistic optimist accepted his medical situation from the start and spoke of his humor despite his unfavorable prognosis:

“*Well, I’m pretty easy. Look, I’m not the only one who has cancer. Yes, we have to make do with what we have. Humor is the most important thing. Yes, of course, you can sit in the corner and think gosh, I have cancer (* . . . *). Yes, why me? Yes, why not someone else? [E1]*”

The professionals told of their professionalism while supporting employees who participated of their own free will. Depending on their experience as a professional, they seemed to rely on their expertise. This might set the scene for the support to be given:

“*I just handled it differently, treated it as a guideline. And I thought: well, I will see if it is appropriate. But I’ve been in the business for so many years, I can also vary it a little bit. [P4]*”

#### 3.4.2 Medical situation (receivers)

Due to different cancer diagnoses, prognoses and length of treatment, the physical and mental condition was something to keep in mind. The stories revealed that the conditional differences experienced could have an impact on the progress to be made.

“*Exercise does help in physical recovery. It also aids in mental recovery. But it is not a guarantee that you can get back to work. [P1]*”

#### 3.4.3 Environmental support (receivers)

The interviewed employees referred to various aspects of support in the private environment and employer support. The majority was grateful for the support received from their family and friends, although they might spare them details out of concern for them.

“*Some things you never discuss or say to your friends or family members. Because it is something heavy. This was just a very safe space, where you could just tell your whole story. That was very nice. [E6]*”

The support from the workplace ranged from a little to a lot of understanding and cooperation. The professionals were also aware of the employer’s concerns in the event of a cancer diagnosis and tried to advise him or her:

“*Often, an employee is at a loss what to do. But the employer is completely at a loss*! *Because he wants to understand and be empathetic, but he also just has a business problem. That is where we often compromise in between. Like* ‘*yes, you can put the business first. Then you do have an employee who will become ill in a few weeks. And then it costs so much each day*’*. [P1]*”

The meetings, together with the social worker (‘three talks’), often proved to be a solution here. It helped address the employer’s concerns. It also helped to explain better how cancer recovery is progressing, and how cancer can delay preparation for returning to work.

### 3.5 Value of the CWS regarding RTW

#### 3.5.1 Return

From the interviewed employees we heard that they felt strengthened by the support. That it could help them to return to work earlier.

“*Yes, I have personally experienced it as a success. The guidance, everything that has been there. I was very happy with that. That I recovered faster and was able to get back to work faster. And that I did not end up in a kind of self-pity. [E13]*”

#### 3.5.2 No return

For some employees the future remained uncertain. They felt motivated to return to the workplace, but medical reasons prevented them from doing so.

“*Then, when we really started to build up a bit, it came back. So yes, then you start all over again. That is actually what happened every time. [E1]*”

#### 3.5.3 Reflection

The employees explained that they had learned to put things in perspective better, which might lead to a more open-minded and positive attitude towards life. Together with the handles they received to cope with obstacles, the employees might look into the future with confidence. Because they felt able to set the boundaries again, some thought about devising new priorities. The employees said that they learned a lot during the sessions anyway and that the CWS in particular created more awareness.

“*Well, I thought it was really additional support. It makes you more aware of how you feel. What you could do, what you would or wouldn*’*t like, or what you don’t want. [E10]*”

The professionals also reflected on the benefits of the CWS:

“*For myself too, as a professional, I really found it of added value. The kind of questions and assignments, I personally think it is a good offer. I thought it was a very nice training and I have benefited a lot from this guidance. I learned a lot from that myself. I also sometimes use parts for other clients. [P3]*”

## 4 Discussion

In this study, we aimed to understand how the Cancer & Work Support (CWS) was experienced by employees with cancer, and by RTW professionals who provided the support. In addition, we wanted to gain insight into when and how the support worked for employees and professionals. From the interviews, we identified two characteristics of a human-centered and tailored support. One aspect related to ‘Involvement’, with regard to the form (‘how’); the second to ‘Approach’, with regard to the content (‘what’). Four themes were covered: open connection and communication; recognition and attention (‘how’); guiding awareness and reflection; and providing strategies to deal with the situation (‘what’). Furthermore, we saw some variation in the experiences, based on personal, medical and environmental differences. The latter corresponds to the general finding that individual characteristics need to be considered, when deciding if and when to return to the workplace [28].

### 4.1 Aims of the CWS

At the start, the CWS aimed to prevent the development of depression and anxiety; to enhance the confidence of patients in their return to work and to support recovery-enhancing behavior including perseverance when returning to work. The findings show indeed that professional help may be useful in reducing symptoms of depression or anxiety, by giving individuals the opportunity to talk freely and safely about their feelings and concerns. The patients were given the time needed for their return to work or to extend working time. Moreover, healthy behavior (e.g. exercising) was a topic at the end of every session. The employees mentioned that they were aware of their reduced energy levels and that they had learned to deal with it. Shaw and colleagues [29] found that physical exercise provided positive effects on wellbeing and was essential for workability. Although we know that twelve of the fourteen employees returned to work, we cannot conclude whether and how the CWS contributed to workability and/or work resumption in a meaningful way for both the employee and the employer. However, we can perhaps agree with the findings of Dorland et al. [30] that reducing symptoms of depression and fatigue and supporting workability can help improve work functioning over time.

### 4.2 Human centered and tailored

The CWS was experienced as human centered. This concept is widely used in business [31] and has some overlap with CWS since the method is developed on the basis of understanding people’s needs and behavior. After all, the CWS was theoretically founded (e.g. on social learning theories) and based on positive experiences of the JOBS program [16] that has been applied to several different groups experiencing ‘transition in life’ such as from school to work [32], from work to work [33], from work to pension [34] and from sick leave to return to work [35]. The principle of change in this transition underlying the JOBS program is creating mastery experiences thereby enhancing self-efficacy and the ability to deal with obstacles and setbacks [17] in safe surroundings, i.e. human centered.

The RTW professionals tailored their support to the needs of the client, based on their expertise as a professional counselor. The social workers, for instance, are used to providing support in case of social problems. For the reintegration coaches the skill and resources module seemed to coincide more with their professional skills. Nevertheless, the professionals were trained beforehand in disease coping and skills protocols, during two refresher-training days. One pitfall might be that they relied on their experience while providing the CWS, meaning that they had to depart from the tight protocol to tailor the program. Did they work sufficiently according to the new method, or did they provide a form of ‘care as usual’?

However, according to the professionals, an important difference with ‘care as usual’ was that the participant employees of the study did not request assistance but were made aware of the existing new way of supporting employees with cancer by the occupational physician. In this way, CWS can be regarded as supply-driven assistance rather than demand-driven help. A second difference was that the CWS was a new and full program, including career tools (skills, resources) as well.

### 4.3 Involvement and approach

If we look at the way in which the RTW professionals were involved in the CWS, we think we see a comparison with the concept of ‘attentiveness’ (in elderly care) from Klaver and Baart [36] and the concept of ‘concernful involvement’ from Yanchar [37]. ‘Attentiveness’ can create a space in which good relationships may arise. This concept stems from the Theory of Presence (ToP) [38], which was developed in the Netherlands in 2011. Healthcare professionals, especially in the fields of hospital and elderly care, should have learned since then how to be ‘present’, and how to connect to the needs of patients. Acknowledgment and being open in a professional caring relationship seem to be needed to ‘being there for someone’, in order to give people the opportunity to show themselves and let them feel they are seen [39]. ‘Concernful involvement’ refers to the recognition that both parties (employees and professionals) are involved in making sense of a world “in which people, objects and events matter” [p.4 in 40]. It is about giving meaning and reflection. Based on our findings, we believe that a good mutual relationship in a trusted open atmosphere may contribute to a better reception of support. Leslie et al. [41] found that a trusting relationship promotes engagement and better collaboration in healthcare settings.

With regard to the open atmosphere during CWS, Haugli and colleagues [42] confirmed that being seen, heard and taken seriously by ‘work and health’ professionals is one of the most valued elements of the RTW process. Moreover, people on long-term sick leave perceive awareness and resources, as well as employer support, to be valuable [42]. We found that the support provided created increased awareness. The employees were given a chance to reflect on their feelings, decisions and actions in an attentive and safe environment. Moreover, they learned how to manage their concerns, which helped them to regain their self-confidence. Together with employer understanding and recognition of their vulnerability, which can be increased or decreased in the workplace [8], this was felt to be an important step forward in preparing their RTW.

The results showed that the providers’ professionalism during the CWS program was highly appreciated by the employees. Which indicates that satisfactory RTW support after cancer cannot be provided by just anyone. Professional competences are important in developing trust [43].

While mentally preparing for RTW, cancer survivors may feel insecure and vulnerable. Many of their inner thoughts and considerations can only and should therefore only be discussed in a safe environment [8]. Similarly, MacLennan et al. [40] pointed out the urgency of receiving support from healthcare professionals. In their study, they found that women with breast cancer are making decisions about workability; they rethink the meaning of work and are in need of professional advice [40]. We do not know whether these findings can be directly generalized to all cancer types, but adequate communication skills and a good relationship seem to be of great importance.

### 4.4 Communication with the workplace

The three-way discussions were held to stay in good contact with the employer and discuss possible RTW options, if desirable. These discussions during sickness-absence have proved to be helpful [44]. In a study among employers, communication with absent employees was found to be crucial. Different communication styles were needed during the consecutive stages in the RTW process: from the moment of disclosure, during sickness absence, RTW planning, until the actual return [14]. Recently, Yagil and Cohen [45] suggested the need for guidelines and training programs to support contact and communication in the workplace during absence from work. The participants in the current study talked about the value of the CWS with regard to communication with the workplace. Although we know that good contact with employers can lead to better RTW experiences [46], the research team did not (have the possibility to) ask the employers directly. However, the findings show that the employers assumed their role in the RTW process and most of them were supportive and understanding. During the CWS, the RTW professionals were able to further inform them regarding their concerns and needs, which was very much appreciated.

### 4.5 Strengths and limitations

Based on the interviews with 22 participants, who were very open during the conversations, we saw that the CWS was highly appreciated by professionals and employees. While focusing on what happened during the sessions, we were able to discover the two characteristics of the CWS. The interviews that were rich in content showed us the challenges the participants (employees and professionals) face, each with regard to their own concerns and in their own way. We mainly focused on the employees’ concerns and challenges. The experiences of both employees and professionals were brought together in the results section, to show that both perspectives underline the findings. This way of describing promotes readability and contributes to the trustworthiness and theoretical generalizability of the findings. Together, eight professionals supported about 40 employees with cancer during the CWS. Thus, the global experiences of more than the 14 employees were discussed. Our sample of employees included a variety of age, cancer types and functions. The professionals also varied in age and experience. However, a limitation might be that we could not compare the different experiences of employees of different ages (the majority between 40 and 60 years; only two < 40) or cancer types (50% breast cancer); nor did we examine employees’ medical conditions, cancer severity, and type of treatment.

Knowing that the study was based on a convenience sample, after six interviews with professionals, we additionally searched for two younger and less experienced social workers. We do not know why professionals and/or employees did not respond to the coordinators’ call to participate in the study. We can only assume it might have something to do with workload (professionals) or with a hesitation to talk about cancer again (survivors). Twelve of the 14 employees had earlier returned to work. Perhaps some of the other supported employees preferred to close the uncomfortable cancer episode and just be thankful that they were able to live a ‘normal’ life again [47].

Furthermore, recall bias may have occurred as for some participants, the CWS support was provided three years ago. Concerns about memory are often reported by cancer survivors [48]. The employees did not necessarily follow all three modules, nor did the professionals deliver them. For that reason, no precise statements can be made about the original aims of the CWS. Nevertheless, we discussed some issues regarding feelings, concerns and work resumption. Two types of professionals delivered the CWS: (occupational) social workers and reintegration coaches from two different providers. This might have led to a somewhat different way of working. The disease coping module seemed more familiar to the social worker, whereas the reintegration coaches were more at home with the skills and resources modules. To reduce the differences regarding the coping and skills modules, two-day training sessions were provided.

### 4.6 What this study adds

In the Netherlands, employees and employers have to collaborate during sickness absence and draw up a reintegration plan in collaboration with the occupational physician. With the CWS, employees with cancer are closely supported after treatment. They are supported in accepting their situation gradually and in shaping their new (working) life little by little. In-depth conversations are possible, about more than just work. Not feeling pushed to RTW, skills and competences will be looked at more closely. In the last module, if applicable, resources are mapped. Awareness is thus created.

An important finding is that the way the participants are involved: the open connection and the attention received, can be seen as a condition for being open to the substantive support to be provided. Contact is maintained with the employer and, if the situation allows, he or she is involved in (preparing) the usually gradual return. What provides peace of mind is that employees are given time to recover and at the same time think about (and prepare) their return at a later stage. Without CWS, employees are alone with their concerns and might then feel pushed to return to work (e.g. in the case of an employer who is not understanding) and feel more dependent on employers’ concerns and wishes.

## 5 Conclusion

We found that both deliverers and receivers highly appreciated the human-centered and tailored CWS with regard to preparing for RTW. In particular, knowledge of the two characteristics in the CWS (involvement and approach), should be taken into account when implementing this method (e.g. in occupational health services) or when developing new supportive measures. A good relationship in an open atmosphere can contribute to a better reception for the support provided. Providing strengthening and problem-solving skills in an atmosphere in which individuals feel safe to talk about themselves can bring about a change in behavior [16]. This research shows that not only ‘what’ you do, but also ‘how’ you do it, is important when supporting RTW. In order to experience the benefits of the CWS, it is necessary that experienced professionals deliver the support.

## Ethics approval

The Medical Ethical Vommittee Brabant approved the current study (NL63659.028.17 / P1756).

## Informed consent

All procedures performed in studies involving human interests were in accordance with the ethical standards of the institutional and/or national research committee and with the 1964 Declaration of Helsinki and its later amendments of comparable ethical standards. Informed consent was obtained from all study participants.

## Appendix

*Topic guide EMPLOYEES*

The purpose of this interview is to find out how you experienced the guidance to work, whether it suited your situation, what you benefited from, what you missed.

➢ How is your situation (daily tasks, kind of work). What does work mean to you?
➢ Do you know when you started the Cancer and Work Support (CWS)? Which modules did you follow, why, when and how many sessions?
➢ How did you experience the support? What was good for you? What wasn’t? And why?
➢ To what extent did you need the module ‘coping with cancer’?
➢ What can you tell us about the timing of discussing the topic of work?
➢ Part of the support was meant to make you think about possible bumps on the way to work. Do you remember if you saw bumps (which ones?) and how did that come up?
➢ Three-way conversations (employee, coach, employer) were part of the guidance. The aim was to keep in touch with the workplace and build up mutual trust and understanding. How did your employer support you, if at all?
➢ To what extent where you able to maintain control over the reintegration yourself?
➢ To what extent could you tailor the CWS to your specific situation? If not, how could it be done differently?
➢ What has the guidance given you that you probably wouldn’t otherwise have had?

Is there anything else you would like to share with us? Additions? Hints? Thank you for your cooperation.

*1.2. Topic guide PROFESSIONALS*

We are interested in how the professionals (and employees) experienced this guidance. We want to discover exactly what works or doesn’t work/help and why. The questions are about your concrete experience and not about your opinion.

➢ How did you find out about the Cancer and Work Support (CWS)? Did you volunteer for it, register yourself?
➢ How did you experience the training for this (form of) guidance? Sufficient y/n?
➢ Have you worked with (grief) counseling before? Is it different now?
➢ How many clients have you guided with (parts of) the CWS? How many sessions?
➢ What was your experience with the timing of discussing the topic ‘work’ (for the people you supervised)?
➢ To what extent (and for what reason) did you adapt to the phase someone was in?
➢ Three-way conversations (manager/coach/employee) were part of the guidance. How did this work in practice? Timing, frequency, content, results?
➢ Part of the guidance was giving control regarding the reintegration to the employee. How did this work out in practice?
➢ Which parts of the module ‘Skills’ worked well and met the needs of the employee (in your experience)?

Do you have anything you want to add to what we’ve discussed so far? Thank you!
